# The Acceleration-Speed Profile in Professional Soccer Players: A Comparative Study According to Sex, the Sports Level and the Playing Position

**DOI:** 10.5114/jhk/220252

**Published:** 2026-07-19

**Authors:** Andrés López-Sagarra, Manuel A. Rodríguez-Pérez, Alexis Padrón-Cabo, Andrés Baena-Raya

**Affiliations:** 1 Sport Science Department, Mexican Football Federation, Mexico City, Mexico.; 2 SPORT Research Group (CTS-1024), CIBIS Research Center, University of Almería, Almería, Spain.; 3 Department of Education, Faculty of Education Sciences, University of Almería, Almería, Spain.; 4 University of Vigo, Faculty of Education and Sport Sciences, Pontevedra, Spain.; 5 Department of Medicine, Division of Endocrinology & Einthoven Laboratory for Experimental Vascular Medicine, Leiden University Medical Center, Leiden, Netherlands.

**Keywords:** competition, football, monitoring, performance, team sports

## Abstract

This study aimed to compare maximum acceleration and speed capabilities in professional soccer players, both derived from the acceleration-speed (AS) profile (theoretical maximum acceleration [A_0_] and speed [S_0_]) and direct assessment (maximum acceleration [Max_Acc_] and speed [Max_Speed_]), according to both (i) sex and the sports level, and (ii) the playing position. A total of 737 professional women and men soccer players were monitored during 5–10 official matches. Data were collected using 18-Hz GPS devices to derive AS profiles and directly measure acceleration and speed output. Across all playing levels, professional men soccer players demonstrated significantly (p < 0.01) higher values than female players for A_0_ (d = 1.14–1.87), S_0_ (d = 3.88–4.82), Max_Acc_ (d = 0.57–1.22), and Max_Speed_ (d = 2.46–2.81). Likewise, players of higher sports levels showed superior output than their lower counterparts (First Men’s Division > Second Men’s Division > Under 20 Men’s Division; p < 0.05). Position-specific analysis revealed that midfielders had significantly (p < 0.05) lower Max_Speed_ values than other positions such as full-backs (d = 0.63–1.17), wide midfielders (d = 0.82), and forwards (d = 0.67–1.06) in both the First and Second Divisions. Conversely, wide midfielders in the Under 20 Men’s Division exhibited significantly (p < 0.05) higher A_0_ values than central defenders (d = 0.78). These findings highlight the importance of conducting sex- and position-specific analyses of the AS profile to effectively monitor acceleration and speed capacities across different sports levels in professional soccer.

## Introduction

Soccer is a multidirectional team-sport characterized by short periods of high-intensity activity (e.g., sprints, accelerations, decelerations, and change of directions) interspersed with longer periods of low-intensity activity (Bangsbo et al., 2006). In the last decade, time-motion analysis revealed that the number of high-intensity actions and high-intensity running distances have increased during professional soccer matches (Lago-Peñas et al., 2023). Thus, professional men players typically execute about ~91–119 high-intensity accelerations (>2.5–3 m/s^2^) and ~16–27 sprints (>6.6–7 m/s), while women players perform about ~41–75 high-intensity accelerations (>2.5–3 m/s^2^) and ~8–15 sprints (>5–5.8 m/s) during a game (Panduro et al., 2022; Pérez Armendáriz et al., 2024). Likewise, a higher sports level has been associated with increased sprinting distances and acceleration frequency compared to lower-level players and competitions (Coso et al., 2020; Haugen et al., 2020). Specifically, playing position analyses indicate that both men and women fullbacks (FBs), wide midfielders (WMFs), and forwards (FWs) exhibit higher sprinting distances and acceleration rates during soccer matches (Ingebrigtsen et al., 2015; Oliva-Lozano et al., 2020). Given the potential of high-intensity actions to significantly affect match outcomes (Caldbeck and Dos’Santos, 2022; Martínez-Hernández et al., 2023), it is crucial to implement monitoring and data-driven decisions to effectively manage these capabilities in soccer.

Maximal speed (Max_Speed_) and acceleration (Max_Acc_) are key metrics for monitoring and managing external loads in soccer (Coso et al., 2020; Dalen et al., 2016). Effective load management can help reduce the risk of non-contact injuries and improve players' ability to perform high-intensity actions such as scoring, dribbling or defending, where the ability to accelerate rapidly (i.e., Max_Acc_) and reach key spaces ahead of opponents (i.e., Max_Speed_) is often decisive (Mendiguchia et al., 2015; Morin et al., 2015). Traditionally, these maximal capacities have been assessed using timing gates or radar devices (Haugen et al., 2020; Haugen and Buchheit, 2016) during testing sessions. However, this setting entails considerable organization and preparation, adding physical and mental loads on players (Morin et al., 2021). Such complexity may hinder continuous monitoring during in-season periods. To overcome this limitation, Morin et al. (2021) recently proposed the acceleration-speed (AS) profile which is constructed by determining the highest acceleration efforts across a range of speed from global positioning system (GPS)-derived training or competition data. This AS profile is well summarized through the theoretical maximal acceleration ([A_0_] y-intercept of the AS linear relationship) and the theoretical maximum speed ([S_0_] x-intercept of the AS linear relationship) and provides insights similar to those obtained from sprint force-velocity (F-V) profiling via isolated sprint tests (Alonso-Callejo et al., 2023). Since valid AS profiles can be obtained from covering a wide range of velocities (from 20% to 95% of an individual's maximum velocity) (Clavel et al., 2022), this approach allows coaches to monitor their players’ physical performance during both training sessions and competition.

The AS profile has proven to be a differentiating factor according to the playing position (Alonso-Callejo et al., 2022; Benhassen et al., 2025; Garrosa et al., 2025), the microcycle day (Alonso-Callejo et al., 2022), and the season period (López-Sagarra et al., 2022) in professional soccer. In professional youth men soccer players, Cardoso et al. (2024) found that the AS profile could discriminate between players’ S_0_, despite the consistent A_0_ values and acceleration-speed slopes observed across various age groups throughout the season (Cardoso et al., 2025). However, neither of these studies accounted for differences by playing position. Additionally, reference values for the AS profile have also been recently established for professional senior (López-Sagarra et al., 2022; Morin et al., 2021), youth men (Cardoso et al., 2024), and senior elite women soccer players (Cormier et al., 2023). The application of reference values across different sports levels, playing positions, and sex can enhance the monitoring and management of players’ physical status (Clavel et al., 2023), talent identification and long-term players’ development (Cardoso et al., 2024). However, the current body of research contributing to these normative datasets remains limited, and the broad spectrum of A_0_ and S_0_ values available presents challenges for practitioners aiming to implement these benchmarks effectively.

Therefore, the main objective of this study was to compare the maximum acceleration and speed capabilities in professional soccer players, using both the AS profile (A_0_ and S_0_) and direct assessments (Max_Acc_ and Max_Speed_), according to (i) sex and the sports level, and (ii) the playing position. Based on previous studies, we hypothesized that (i) men players across all sports levels would exhibit greater values than women players, and that players of higher sports levels would outperform those at lower levels (Cardoso et al., 2024; Cormier et al., 2023; López-Sagarra et al., 2022), and (ii) players in wide positions such as the FB and the WMF would exhibit higher acceleration and speed values, while central defender (CD) and midfielder (MF) positions would show the lowest values (Alonso-Callejo et al., 2022; Oliva-Lozano et al., 2020).

## Methods

### Participants

Seven hundred thirty-seven professional men (n = 574) and women (n = 163) soccer players participated in this study. Players from different positions (CD, FB, MF, WMF and FW) belonged to 72 different teams from four categories (18 teams per category) of the Mexican Soccer League: First Men’s Division (n = 189; CDs = 49; FBs = 44; FWs = 25; MFs = 57; WMFs = 14; age: 27.2 ± 4.5 years), Second Men’s Division (n = 171; CDs = 45; FBs = 32; FWs = 18; MFs = 54; WMFs = 23; age: 25.1 ± 4.4 years), Under 20 (n = 214; CDs = 64; FBs = 44; FWs = 25; MFs = 62; WMFs = 19; age: 19.0 ± 1.8 years) and the First Women’s Division (n = 163; CDs = 39; FBs = 31; FWs = 26; MFs = 55; WMFs = 12; age: 25.9 ± 4.6 years). Goalkeepers were excluded from the subsequent analysis due to the differences in external load demands. Additionally, participants had to meet the following criteria: (i) players had to play at least 5 matches (ii) and play the whole duration of the game. Data collection for this retrospective analysis was conducted as part of the routine training regimen of each team, under usual technical and medical oversight. Thus, no specific interventions were necessary for this study, which adhered to the principles outlined in the Declaration of Helsinki (World Medical Association, 2013). This study was approved by the Institutional Review Board of the Bioethics Commission of the University of Almería, Almería, Spain (protocol code: UALBIO2024/017; approval date: 12 July 2024).

### Design

A retrospective longitudinal observational research design was used to study the AS profiles of professional soccer players. All players completed a 4- to 5-week preseason period before the start of competition. All men’s categories began simultaneously, whereas the First Women’s Division started two weeks later. To minimize potential bias due to early-season variability and fixture congestion, particularly in the First, Second, and Under 20 Men’s Divisions, the first month of competition was excluded from the analysis. Thus, data collection spanned an 8-week period from September to October, corresponding to the second and third months of the 2022–2023 competitive season. Players in the Second and Under 20 Men’s categories participated in a maximum of 8 official matches, while those in the First Men’s and First Women’s Divisions played up to 10 matches. No external interventions were introduced, allowing for natural monitoring of each player’s AS profile under typical competitive conditions throughout the season.

### Methodology

All players used the WIMU Pro 18-Hz GPS tracking system (Hudl, Lincoln, US) for uniform data collection during soccer matches. This system had demonstrated both reliability and validity for time-motion analysis in soccer, showing minimal biases in mean velocity (1.18 ± 1.32 km/h) and mean distance (2.32–4.32 m), with robust intraclass correlation coefficients exceeding 0.93 (Bastida-Castillo et al., 2018). It had also received FIFA certification for time-motion analysis (Haycraft and Aughey, 2022) after rigorous testing procedures (Oliva-Lozano and Muyor, 2022). During monitoring, an average of 12.1 ± 3.2 satellites were consistently connected, and the horizontal geometric dilution of precision was maintained at an ideal level of 0.59 ± 0.18, ensuring data quality (Malone et al., 2017; Rico-González et al., 2020). To avoid inter-unit variability error, all players used the same GPS unit during training sessions and matches (Malone et al., 2017). Specifically, the devices were placed in the vest pocket on the player's upper back before each game and collected by the team staff after matches.

Raw acceleration and speed data were extracted using SPro (version 990) software (Hudl, Lincoln, US) and uploaded to the data storage cloud provided by the manufacturer (WimuCloud). A dataset containing between 300,000 and 500,000 AS points per player was created to calculate a single individual AS profile using match data collected over an 8-week period. Following strictly the method proposed by Morin et al. (2021), only positive accelerations between 3 m/s and the player's maximum speed were considered. This range of values was divided into intervals of 0.2 m/s. Outliers (outside the 95% of confidence interval) were removed to improve the accuracy of the results and the two highest acceleration values within each interval were selected. Simple linear regression was then used to fit the two maximum AS points per interval (e.g., 3, 3.2, 3.4 m/s, etc.). Finally, the A_0_ (X = 0 and y-intercept) and S_0_ (Y = 0 and x-intercept) values were obtained from the linear regression for each player. To ensure the validity of A_0_ and S_0_ values, all players included in the analysis reached maximum speed values and maximum acceleration values covering the entire velocity spectrum (Clavel et al., 2023). RStudio software (R Core Team, 2022) was used for the calculation of the AS profile. The developed code is provided as supplementary material. Max_Speed_ and Max_Acc_ values were directly selected from individual raw data registered during the matches.

### Statistical Analysis

Descriptive characteristics are presented as means and standard deviations (SDs). Statistical analyses were performed using statistical software R (version 4.2.3; R Core Team, 2023). The R package *lme4* (Bates et al., 2015) was used to fit a linear mixed-effects model to analyze differences in the AS profile according to the sports level and sex (First Men’s Division *vs*. First Women’s Division *vs*. Second Men’s Division *vs*. Under 20 Men’s Division), as well as the playing position. Specifically, the sports level and the playing position were fitted as fixed effects, while the team identity was modelled as a random effect. The residuals of the linear mixed model were examined to assess the assumptions of homogeneity and normality, with no evidence of assumption violations. Pairwise comparisons were adjusted using the Bonferroni’s post-hoc test via the R package emmeans (Lenth et al., 2020). Additionally, Cohen´s *d* (*d*) was calculated and categorized as trivial (<0.20), small (0.20 to <0.60), moderate (0.60 to <1.20), large (1.20 to <2.00), and extremely large (>2.00) (Hopkins et al., 2009). The level of significance for all statistical analyses was set at *p* ≤ 0.05. All plots were generated using Python (Python Software Foundation, version 3.10) with the Matplotlib and NumPy libraries.

## Results

Table 1 shows the maximal acceleration and speed capabilities according to sex, the sports level, and the playing position.

**Table 1:** Maximal acceleration and speed capabilities per sex, sports level, and playing position and difference in means.

	Positions	A_0_ (m/s^2^)	S_0_ (m/s)	Max_Acc_ (m/s^2^)	Max_Speed_ (m/s)
First Men’s Division	CD	7.33 ± 0.63	9.64 ± 0.34	6.02 ± 0.54	8.86 ± 0.41
	FB	7.23 ± 0.56	9.62 ± 0.27	5.84 ± 0.50	9.02 ± 0.36^c^
	FW	7.51 ± 0.71	9.64 ± 0.29	6.12 ± 0.58	9.08 ± 0.54^b^
	MF	7.46 ± 0.68	9.56 ± 0.34	5.56 ± 0.46	8.77 ± 0.42^bcd^
	WMF	7.39 ± 0.57	9.73 ± 0.34	5.91 ± 0.59	9.11 ± 0.38^d^
	All	7.37 ± 0.64	9.61 ± 0.32	5.94 ± 0.52	8.91 ± 0.43
First Women’s Division	CD	6.42 ± 0.51	7.92 ± 0.32	5.40 ± 0.61	7.58 ± 0.37
	FB	6.20 ± 0.32	8.01 ± 0.32	5.10 ± 0.44	7.77 ± 0.40
	FW	6.32 ± 0.41	8.03 ± 0.31	5.23 ± 0.50	7.84 ± 0.35
	MF	6.32 ± 0.46	8.02 ± 0.37	5.33 ± 0.55	7.78 ± 0.41
	WMF	6.46 ± 0.34	7.95 ± 0.50	5.29 ± 0.54	7.71 ± 0.52
	All	6.33 ± 0.43	7.98 ± 0.35	5.29 ± 0.54	7.73 ± 0.43
Second Men’s Division	CD	7.02 ± 0.54	9.50 ± 0.33	5.72 ± 0.50	8.76 ± 0.40
	FB	7.17 ± 0.48	9.61 ± 0.27	5.71 ± 0.50	8.95 ± 0.30^f^
	FW	7.31 ± 0.68	9.50 ± 0.30	5.84 ± 0.55	8.92 ± 0.31^e^
	MF	7.12 ± 0.49	9.32 ± 0.34	5.56 ± 0.46	8.55 ± 0.36^ef^
	WMF	7.15 ± 0.59	9.46 ± 0.31	5.73 ± 0.52	8.84 ± 0.31
	All	7.12 ± 0.54	9.46 ± 0.33	5.68 ± 0.50	8.75 ± 0.38
Under 20 Men’s Division	CD	6.79 ± 0.54^i^	9.48 ± 0.33	5.50 ± 0.50	8.64 ± 0.39
	FB	6.92 ± 0.51	9.49 ± 0.27	5.55 ± 0.47	8.79 ± 0.41
	FW	6.96 ± 0.47	9.43 ± 0.29	5.65 ± 0.43	8.88 ± 0.40
	MF	6.87 ± 0.49	9.46 ± 0.31	5.58 ± 0.43	8.65 ± 0.40
	WMF	7.21 ± 0.53^i^	9.51 ± 0.26	5.73 ± 0.45	8.91 ± 0.44
	All	6.89 ± 0.52	9.47 ± 0.29	5.57 ± 0.43	8.71 ± 0.40

A_0_, theoretical maximal acceleration; S_0_, theoretical maximal speed; Max_Acc_, maximal acceleration; Max_Speed_, maximal speed; CD, central defender; FB, fullback; FW, forward; MF, midfielder; WMF, wide midfielder; Significance level = 0.05; ^b^: differences between First Men’s Division MFs and FWs; ^c^: differences between First Men’s Division MFs and FBs; ^d^: differences between First Men’s Division MFs and WMFs; ^e^: differences between Second Men’s Division MFs and FWs; ^f^: differences between Second Men’s Division MFs and FBs; ^i^: differences between Under 20 Division CDs and WMFs

### Sports Level and Sex-Specific Differences

**Figure 1:**
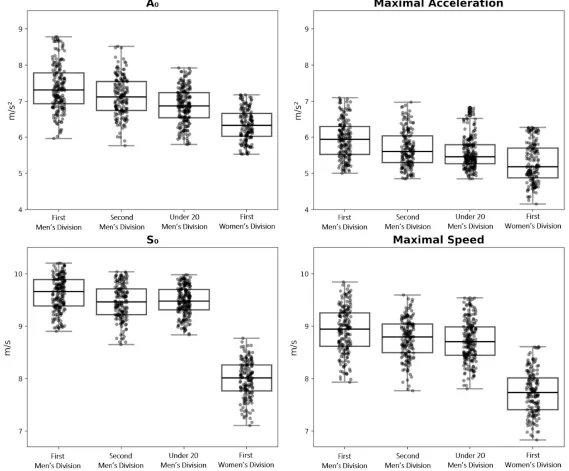
. Comparison of maximal acceleration and speed capabilities according to sex and the sports level.

Figure 1 shows differences in maximum acceleration and speed capabilities according to the sports level and sex. Regarding sex-specific differences, First Women’s Division soccer players presented significantly (p < 0.01) lower values in A_0_ (*d* = 1.14–1.87), S_0_ (*d* = 3.88–4.82), Max_Speed_ (*d* = 2.46–2.81), and Max_Acc_ (*d* = 0.57–1.22) compared to men’s soccer players of all sports levels.

In reference to men’s sports levels, First Division players displayed significantly (*p* < 0.01) higher values in A_0_ (*d* = 0.42; 95% CI = 0.21–0.63), S_0_ (*d* = 0.48; 95% CI = 0.28–0.67), Max_Speed_ (*d* = 0.39; 95% CI = 0.18–0.60), and Max_Acc_ (*d* = 0.50; 95% CI = 0.30– 0.72) compared to their Second Division counterparts. Similarly, First Men’s Division players showed significantly (*p* < 0.01) higher values in A_0_ (*d* = 0.84; 95% CI = 0.64–1.04), S_0_ (*d* = 0.48; 95% CI = 0.28–0.68), Max_Speed_ (*d* = 0.48; 95% CI = 0.28–0.68), and Max_Acc_ (*d* = 0.75; 95% CI = 0.57–0.98) than Under 20 men’s soccer players. In addition, Second Men’s Division players exhibited significantly (*p* < 0.05) greater values in A_0_ (*d* = 0.45; 95% CI = 0.25–0.66) than Under 20 men’s soccer players.

### Playing Position Differences

**Figure 2:**
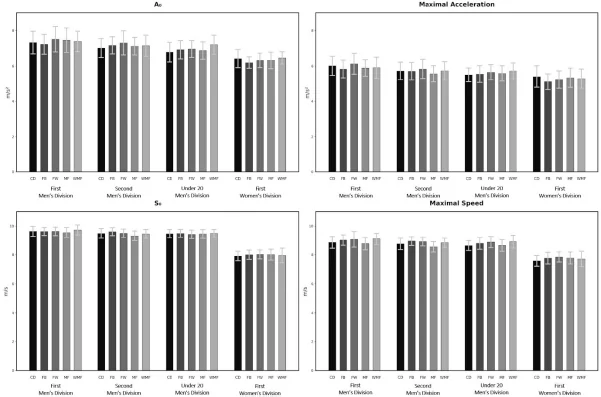
. Comparison of maximal acceleration and speed capabilities according to the playing position.

Figure 2 presents differences in maximum acceleration and speed capabilities, within categories, according to the playing position. In the First Men’s Division category, MFs presented significant (*p* < 0.05) lower Max_Speed_ compared to FBs (*d* = 0.63; 95% CI = 0.21–1.05), FWs (*d* = 0.67; 95% CI = 0.17–1.16), and WMFs (*d* = 0.82; 95% CI = 0.21–1.43). Similarly, Second Men’s Division MF players displayed significantly (*p* < 0.05) lower values than FBs (*d* = 1.17; 95% CI = 0.70–1.65) and FWs (*d* = 1.06; 95% CI = 0.50–1.62) players. Additionally, Under 20 men’s CD players exhibited significantly lower (*p* < 0.05) values in A_0_ compared to WMFs (*d* = 0.78; 95% CI = 0.29–1.27). No significant differences (*p* > 0.05) were observed across playing positions in First Women’s Division players.

## Discussion

This study compared the maximum acceleration and speed capabilities, both derived from the AS profile (A_0_ and S_0_) and direct assessment (Max_Acc_ and Max_Speed_), according to (i) sex and the sports level, and (ii) the playing position in professional soccer players. The main findings of this study indicate that men players across all categories demonstrated, on average, higher values for A_0_, S_0_, Max_Acc_, and Max_Speed_ output compared to women players. Second, acceleration and speed capacities were generally greater in players of higher sports levels, with the exception of S_0_, where Under 20 men players exhibited slightly greater values than those in the Second Men’s Division (Figure 1). Finally, MF players showed significantly lower Max_Speed_ values compared to other positions (FB, FW and WMF) in both the First and Second Divisions, whereas WMFs in the Under 20 Division showed significantly higher A_0_ values than CDs.

Despite the growing body of literature investigating the AS profile in soccer, sex-specific information is still limited. Consequently, training for women players is often derived from men data. In this study, we found that men soccer players from all categories exhibited higher values for A_0_, S_0_, Max_Speed_, and Max_Acc_ compared to women. This is consistent with previous non sex-specific designs that suggested higher values for men (A_0_ = 6.20–8.41 m/s^2^; S_0_ = 9.18–9.47 m/s; Max_Acc_ = 4.5–5.5 m/s^2^ and Max_Speed_ = ~8.4–9 m/s) compared to women soccer players (A_0_ = 6.07–6.47 m/s^2^; S_0_ = 8.11–8.31 m/s; Max_Acc_ = 3.8 m/s^2^ and Max_Speed_ = ~7.4–7.6 m/s) (Alonso-Callejo et al., 2022; Cardoso et al., 2024; López-Sagarra et al., 2022; Morin et al., 2021). Of note, differences in S_0_ and Max_Speed_ were more pronounced compared to those in A_0_ and Max_Acc_, which concurs with the findings of Haugen et al. (2020). They reported greater sex differences in the theoretical maximal velocity compared to the theoretical maximal force obtained during the sprint force-velocity profile. Importantly, sex differences during high-speed tasks have been previously attributed to differences in lower-limb strength, force application, neuromuscular properties, and the contribution of energy systems (Dos’Santos et al., 2018; Spiteri et al., 2014; Tortu et al., 2024). These findings suggest that AS profile data should not be used interchangeably for professional men and women soccer players. Instead, sex-specific data treatment is warranted.

Time motion analysis in soccer matches has shown that players competing at higher levels consistently cover greater sprint distances and perform more accelerations than those at lower sports levels (Sæterbakken et al., 2019). As a result, acceleration and speed capacities have become key performance indicators in professional soccer, given their decisive role in high-intensity actions that can influence the match outcome (Deprez et al., 2015; Haugen et al., 2013). In this context, the present study is the first to analyze the AS profile according to the sports level, thereby limiting direct comparisons with previous research. Notably, our study reported generally superior values of A_0_, S_0_, Max_Acc_ and Max_Speed_ in players of higher sports levels (i.e., First Division > Second Division > Under 20). These findings are consistent with previous literature related to the sprint F-V profile (Devismes et al., 2021; Haugen et al., 2020; Jiménez-Reyes et al., 2019). Specifically, Devismes et al. (2021) found that all variables of the sprint F-V profile were higher in elite men's soccer players compared to those of lower sports levels. Similarly, Jimenez-Reyes et al. (2019) also reported higher values of F_0_, V_0_ and P_max_ in the sprint F-V profile among players of higher sports levels. Consequently, the AS profile emerges as a sensitive method to discriminate between sports levels in soccer similarly to the sprint F-V profile (Baena-Raya et al., 2020; Devismes et al., 2021; Haugen et al., 2020; Jiménez-Reyes et al., 2019). Therefore, the present study may contribute to defining AS profile benchmarks across competitive levels, offering valuable insights for talent identification and the development of individualized training programs.

Previous research suggests position-related disparities in sprint mechanical properties in professional soccer players (Benhassen et al., 2023; Haugen et al., 2020). Overall, FWs and WMFs tend to present higher maximal power and velocity output compared to CDs (Benhassen et al., 2023; Haugen et al., 2020). These findings concur with the results of our study. For the first time, we provide insight into the acceleration and speed capacities of professional soccer players across a broader range of playing positions than previously documented. Specifically, FWs, WMFs and FBs exhibited significantly higher Max_Speed_ values compared to MFs in both First and Second Division players. This could be attributed to the tactical role of FWs, WMFs and FBs who are frequently involved in sprint-intensive phases of play and cover longer distances at higher speeds during matches (Oliva-Lozano et al., 2020). In contrast, MFs displayed lower S_0_ and Max_Speed_ values across men categories, likely due to their tactical role within the game, focusing on passing and ball retention, as well as a higher density of players in central areas of the field (Asian-Clemente et al., 2021). These factors may limit their exposure to high-speed tasks. Of note, no position-related differences were reported among women players. Importantly, only significant differences were noted for A_0_ in Under 20 men players where WMFs exhibited significantly greater values than CDs. Finally, no significant differences were reported for Max_Acc_ within any category, which is consistent with the comparable values observed among playing positions in the theoretical maximal force during sprint acceleration in both professional men and women players. Altogether, our findings confirm the need for position-specific monitoring and training prescription to enhance on-field maximal speed performance and meet the physical demands associated with each playing role.

This study has some limitations that need to be acknowledged. Firstly, women soccer players included in the sample presented only one sports level (First Division); therefore, the present findings cannot be generalized to other competitive levels, which should be addressed in future research. Secondly, the AS profile was individually determined based on official soccer matches during the second and third months of the competitive period. Consequently, the season period, microcycle type periodization and contextual variables (i.e., match location or the quality of the opponent) could influence the match running performance (Douchet et al., 2024; González-Rodenas et al., 2024). Additionally, this research design examined a range of 5–10 soccer matches, what could be considered a relatively small sample size. Nevertheless, this sample size is similar to previous studies that have analysed the AS profile in soccer. Lastly, the sample was composed of one league only, so the results could not be generalized to other soccer competitions. Further studies should attempt to present a larger sample size, different sports levels in women, training sessions and match events to determine the AS profile, and control for contextual variables.

## Conclusions

Professional men soccer players from all categories exhibited greater A_0_, S_0_, Max_Acc_, and Max_Speed_ output compared to women players. Likewise, players of higher sports levels seemed to display greater acceleration and speed output compared to their lower counterparts. In addition, First and Second Division FB, FW and WMF players showed superior Max_Speed_ values compared to other playing positions. Therefore, our current findings suggest strength and conditioning coaches to implement sex- and position-specific analyses of the AS profile to effectively monitor acceleration and speed capacities across different sports levels in professional soccer.

## Supplementary Material

# Reading and filtering data setwd("~/Desktop/AS Profile") data <- read.csv('exampleFile.csv', sep = ';')

# Initial filter: only positive accelerations data_acc <- data[data$acc > 0,]

# Filter by minimum speed of 3 m/s data_spd <- data_acc[data_acc$speed >= 3,]

# Remove outliers: keep only data within the 95% confidence interval of acceleration acc_mean <- mean(data_spd$acc) acc_sd <- sd(data_spd$acc) lower_bound <- acc_mean - 1.96 * acc_sd upper_bound <- acc_mean + 1.96 * acc_sd data_spd <- data_spd[data_spd$acc >= lower_bound & data_spd$acc <= upper_bound,]

# Scatter plot library(ggplot2); ggplot(data = data_spd, mapping = aes(x = speed, y = acc)) + geom_point() + scale_x_continuous(limits = c(0,9), breaks = 0:9)

# AS Profile cuts <- seq(from = 3, to = max(data_spd$speed) + 0.2, by = 0.2) data_spd$cuts <- cut(data_spd$speed, breaks = cuts, right = FALSE, include.lowest = TRUE)

library(dplyr) data_profile <- data_spd %>% group_by(cuts) %>% top_n(2, acc)

# Final plot with highlighted points and regression line ggplot(data = data_profile, mapping = aes(x = speed, y = acc)) + geom_point(data = data_acc, mapping = aes(x = speed, y = acc), color = 'grey', alpha = 0.3) + geom_point(data = data_spd, mapping = aes(x = speed, y = acc), color = 'black') + geom_point(color = 'red') + scale_x_continuous(limits = c(0,9), breaks = 0:9) + stat_smooth(method = lm, se = TRUE, level = 0.99)
